# Identification of prognostic and bone metastasis​‐related alternative splicing signatures in mesothelioma

**DOI:** 10.1002/cam4.3977

**Published:** 2021-05-26

**Authors:** Runzhi Huang, Zixuan Zheng, Sijia Liu, Penghui Yan, Dianwen Song, Huabin Yin, Peng Hu, Xiaolong Zhu, Zhengyan Chang, Yihan Liu, Juanwei Zhuang, Tong Meng, Zongqiang Huang, Jie Zhang

**Affiliations:** ^1^ Department of Gynecology Shanghai First Maternity and Infant Hospital Tongji University School of Medicine Shanghai China; ^2^ Division of Spine Department of Orthopedics Tongji Hospital affiliated to Tongji University School of Medicine Shanghai China; ^3^ Tongji University School of Medicine Shanghai China; ^4^ Department of Orthopedics The First Affiliated Hospital of Zhengzhou University Zhengzhou China; ^5^ Department of Orthopedics Shanghai General Hospital School of Medicine Shanghai Jiaotong University Shanghai China; ^6^ Department of Pathology Shanghai Tenth People's Hospital Tongji University School of Medicine Shanghai China

**Keywords:** alternative splicing, mesothelioma, metastasis, prognosis

## Abstract

Mesothelioma (MESO) is an infrequent tumor derived from mesothelial cells of pleura, peritoneum, pericardium, and tunica vaginalis testis. Despite advancement in technologies and better understanding of tumor progression mechanism, the prognosis of MESO remains poor. The role of alternative splicing events (ASEs) in the oncogenesis, tumor metastasis and drug resistance has been widely discussed in multiple cancers. But the prognosis and potential therapeutic value of ASEs in MESO were not clearly studied by now. We constructed a prognostic model using RNA sequencing data and matched ASE data of MESO patients obtained from the TCGA and TCGASpliceSeq database. A total of 3,993 ASEs were identified associated with overall survival using Cox regression analysis. Eight of them were finally figured out to institute the model by lasso regression analysis. The risk score of the model can predict the prognosis independently. Among the identified 390 splicing factors (SF), HSPA1A and DDX3Y was significantly associated with 43 OS‐SEs. Among these OS‐SEs, SNX5‐58744‐AT (*p* = 0.048) and SNX5‐58745‐AT (*p* = 0.048) were significantly associated with bone metastasis. Co‐expression analysis of signal pathways and SNX5‐58744‐AT, SNX5‐58745‐AT was also depicted using GSVA. Finally, we proposed that splicing factor (SF) HSPA1A could regulate SNX5‐58744‐AT (R = −0.414) and SNX5‐58745‐AT (R = 0.414) through the pathway “Class I MHC mediated antigen processing and presentation” (R = 0.400). In this way, tumorigenesis and bone metastasis of MESO were controlled.

## Graphical abstract

Despite advancement in technologies and better understanding of tumor progression mechanism, the prognosis of Mesothelioma (MESO) remains poor. Alternative splicing events (ASEs) are widely reported in the tumorigenesis, metastasis and drug resistance of multiple cancers. Based on the results of this study, we proposed that splicing factor (SF) HSPA1A could regulate SNX5‐58744‐AT (R = ‐0.414) and SNX5‐58745‐AT through “Class I MHC mediated antigen processing and presentation” pathway and subsequently impact tumorigenesis and bone metastasis of MESO.

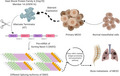

## 1 INTRODUCTION

Mesothelioma is an infrequent cancer with poor prognosis, the average survival time of malignant mesothelioma patients is 12 to 17 months.^1^, ^2^ Generally, mesothelioma originates from the mesothelial cells of pleura and peritoneum.^3^ Exposure of asbestos is an exclusive risk factor and effusion is always the main clinical symptom.^4^, ^5^ There is no uniform treatment for mesothelioma. Although surgical resection at early stage may be an effective method, the prognosis for patients is still poor, with a high metastasis rate to contralateral pleura and lung, liver, bone and brain via direct invasion, lymphatic and hematogenous routes.^6^, ^7^, ^8^ Thus, the pathogenic and metastatic mechanism need to be further explored.

In the basic biological process of eukaryotic organism, such as cell development, cell differentiation and response to environmental factors, alternative splicing (AS) plays vital roles. AS is a common regulatory mechanism and the giant majority, more than 95% of mRNA, are subjected AS.^9^, ^10^ In the meantime, aberrant alternative splicing events (ASEs) are frequently detected in several pathologies including cancers.^11^ Although various studies have shown the mechanisms of AS in the tumorigenesis and metastasis, including our previous ones, their function and regulatory mechanism in mesothelioma remained to be elucidated.^11^, ^12^, ^13^, ^14^

In this study, we plan to explore the mechanism of ASEs in the cancer prognosis and bone metastasis of mesothelioma patients and construct a novel clinical prognostic model based on the ASEs.^15^ Moreover, we proposed a mechanism that splicing events SNX5‐58744‐AT and SNX5‐58745‐AT were regulated by splicing factor HSPA1A. And subsequently impact tumor progression, bone metastasis and poor prognosis.

## 2 METHODS

### 2.1 Data sources

RNA‐seq data were extracted from the TCGA Data Portal (https://tcga‐data.nci.nih.gov/tcga/).^16^, ^17^ Corresponding entries of ASEs were matched from the TCGASpliceSeq database (https://bioinformatics.mdanderson.org/TCGASpliceSeq/),^17^ and 84 cases were eventually enrolled into this study. The ASEs were generally divided into seven subtypes (ES: exon skip, AP: alternate promoter, ME: mutually exclusive exons, AT: alternate terminator, RI: retained intron, AA: alternate acceptor site, AD: alternate donor site).^18^ Each ASEs were allocated a specific annotation which combined the gene name, splicing type, and the ID number in the TCGASpliceSeq database (AS ID). For instance, in this annotation term “DUT‐30485‐AP”, DUT is the gene name, 30485 is the AS ID, AP is the splicing event. To initially examine our hypothesis, external MESO data were extracted from the Gene Expression Omnibus database (https://www.ncbi.nlm.nih.gov/geo/) (GSE number: GSE112154, GSE12345 and GSE99070).^19^, ^20^, ^21^

### 2.2 Identification of OS‐SE

To filtrate prognosis associated ASEs, univariate Cox regression analysis was conducted. And the results were shown using the upset plot and volcano plot. Seven bubble plots were also generated to show the top 20 OS‐SEs of each splicing pattern, the predictive values of ASEs were described by the color and size of bubbles.

### 2.3 Construction of the predictive model

Top 20 OS‐SEs were filtered as potential features of the prognostic model using the Lasso regression model. Multivariate Cox regression model was subsequently performed to evaluate the regression coefficient of each OS‐SE screened by Lasso regression based on β value. And risk score was calculated by this formula:

i=1nβi×PSI

Samples were divided into two groups using the median risk score. To compare the survival between two groups, Kaplan–Meier survival analysis was subsequently performed. The accuracy of the model was assessed by the area under ROC curve. Expression heatmap and scatterplot were generated to visualize the change trend of survival with risk score. Multivariate Cox regression analysis was also applied modifying by age, gender, pathological stage and TNM stage to estimate if the risk score was an independent prognostic factor.

### 2.4 Construction of the potential regulatory network

Data of 390 SFs were recaptured from the SpliceAid2 database.^22^ Regulation pairs of OS‐SEs and SFs were screened according to results of Pearson correlation analysis, the criteria was the absolute value of correlation coefficient >0.350 and a P value < 0.001. The network was subsequently plotted by Cytoscape (3.7.1).^23^ In the network, OS‐SEs and SFs were respectively defined as ellipses and arrows, low and high risk OS‐SEs as purple and red, negative and positive regulations of OS‐SEs and SFs as green and red lines.

### 2.5 Identification of bone/distant metastasis‐related OS‐SEs

To further confirm bone/distant metastasis related OS‐SEs in the network, non‐parametric test was conducted. Overlapped OS‐SEs associated with bone metastasis and SFs were shown using venn diagram.

### 2.6 Co‐expression analysis and functional enrichment analysis

Gene Set Variation Analysis (GSVA) was conducted to figure out most enriched pathways.^24^ And prognosis‐related pathways were subsequently filtrated using the univariate Cox analysis. To investigate mechanism of splicing events, co‐expression analysis was also performed on these pathways and selected ASEs.

### 2.7 External validation

To minimize bias, multiple databases including LinkedOmics,^25^ UALCAN,^26^ the Gene Expression Profiling Interactive Analysis (GEPIA),^27^ PROGgeneV2,^28^ cBioPortal for Cancer Genomics ^29^, ^30^ and Cancer Cell Line Encyclopedia (CCLE)^31^ were used to obtain expression level of key biomarkers at clinical and genome levels. Additionally, data from GEO were also utilized to further verify the correlation of key genes with MESO.

### 2.8 Data analysis

All statistical analyses were implemented using R version 3.5.1. (Institute for Statistics and Mathematics, Vienna, Austria; www.r‐project.org) (Package: impute, rms, ggplot2, UpSetR, glmnet, forestplot, preprocessCore, survivalROC, survminer, beeswarm). Bilateral *p* < 0.05 were defined as statistically significant.

## 3 RESULT

### 3.1 Overview of ASEs in MESO

**FIGURE 1:**
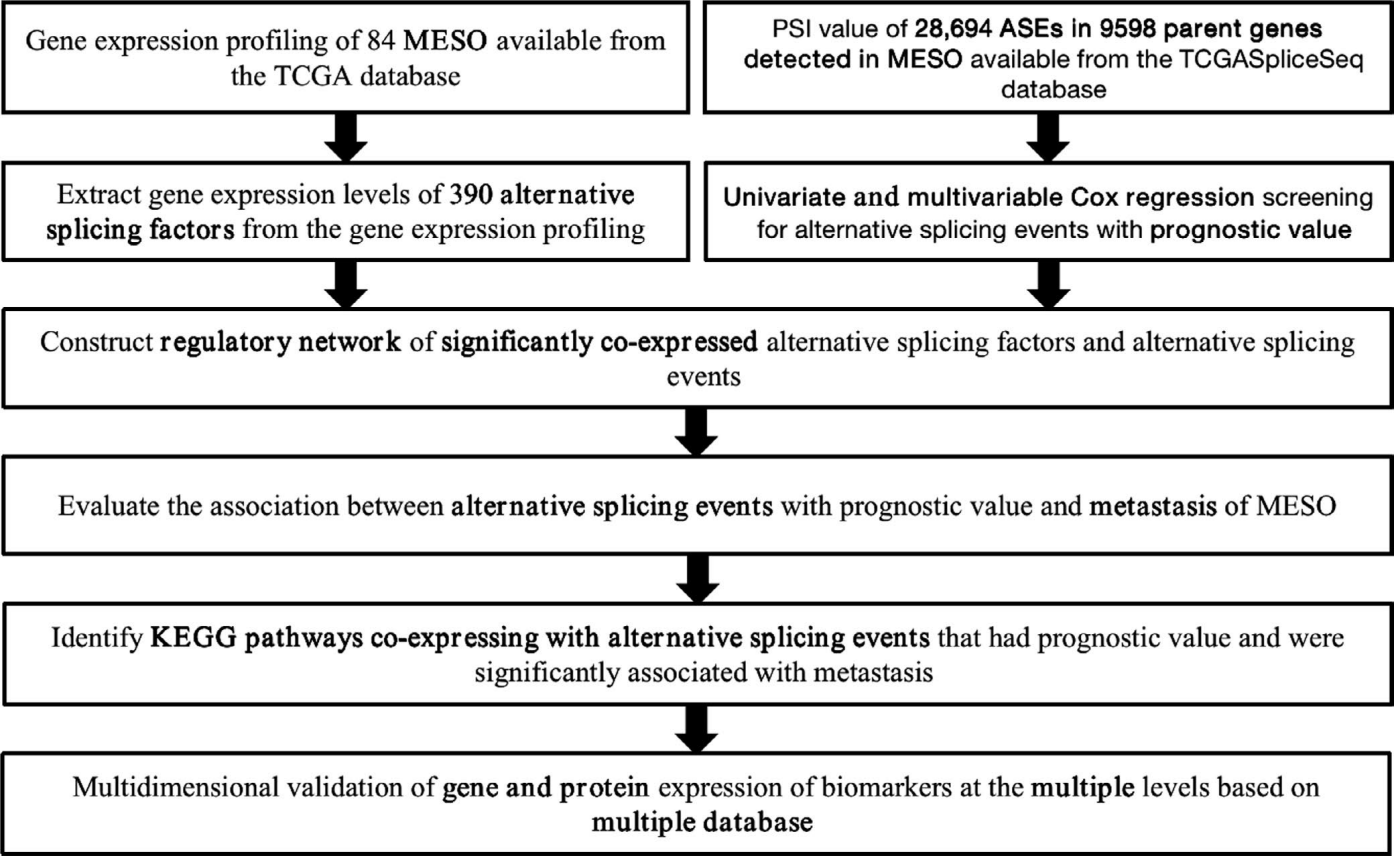
Flowchart of this study

**FIGURE 2:**
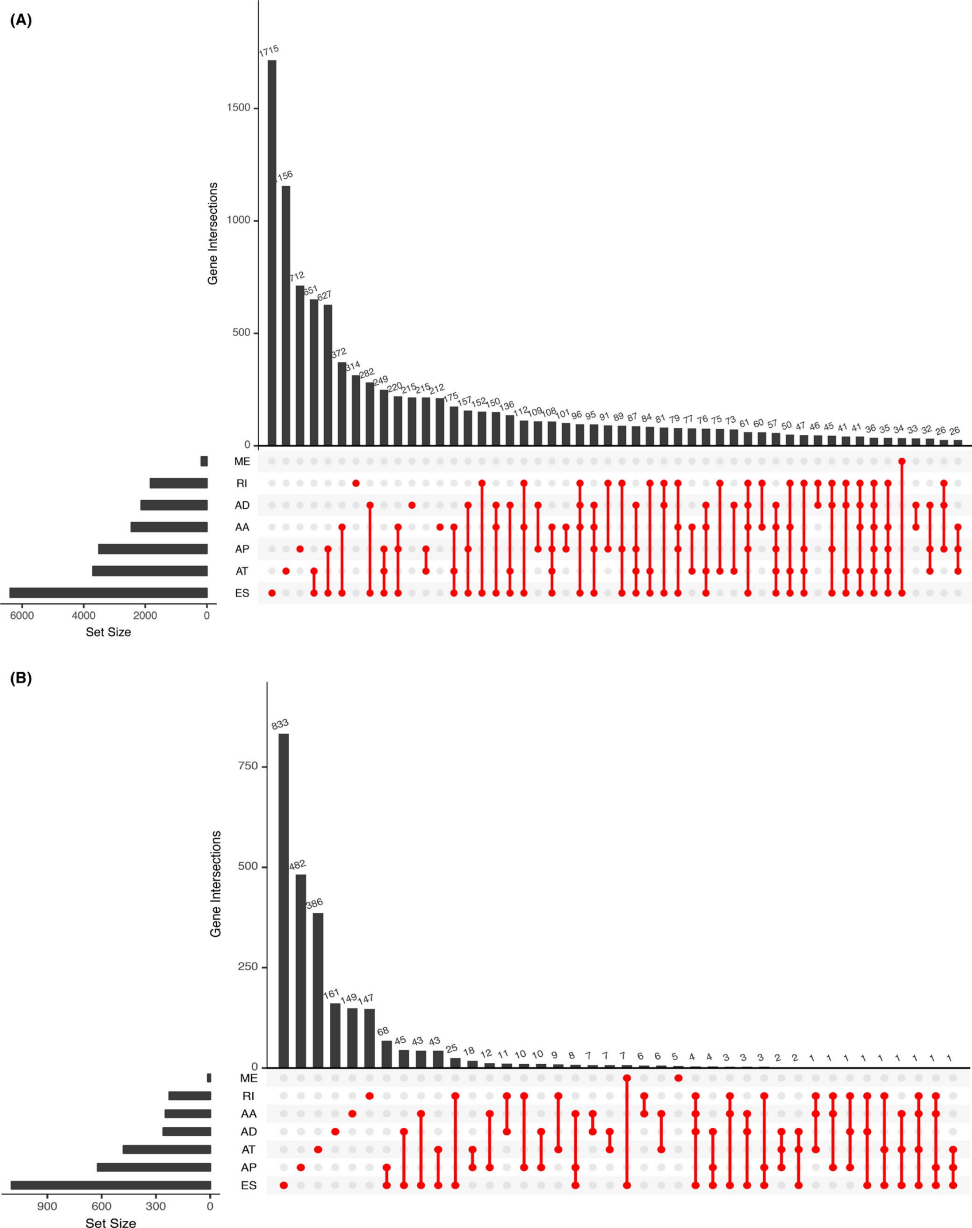
The Upset plots of ASEs and parent genes: (A) The number of ASEs in different types of splicing patterns; (B) The number of OS‐SEs in different types of splicing patterns

**FIGURE 3:**
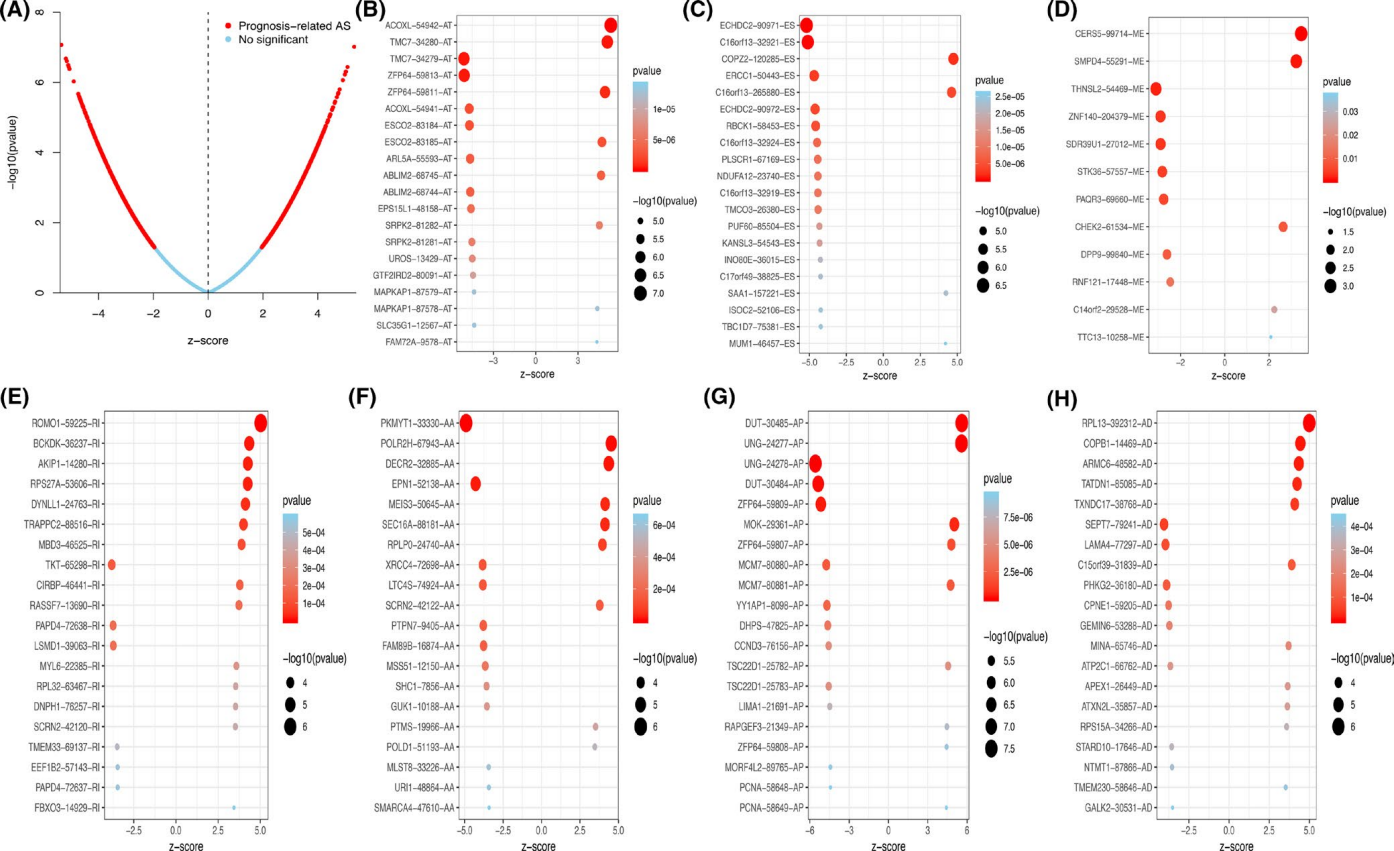
Top 20 OS‐SE in different splicing patterns. (A) The volcano plot showing the prognosis‐related and no significant ASEs respectively; (B‐H) Top 20 OS‐SEs in seven splicing patterns. Abbreviation: AA, alternate acceptor; AD, alternate donor; AP, alternate promoter; AT, alternate terminator; ES, exon skip; ME, mutually exclusive exons; RI, retained intron

The schematic figure of this integrated study was shown in Figure 1. The clinical information was summarized in Table 1. From the TCGA dataset, 84 cases were derived including 25 with metastasis and 59 without metastasis. We obtained information of 28,694 authentic ASEs in 9,598 genes of the MESO cohort: 11,384 ES events in 6,240 genes, 5,540 AP events in 3,012 genes, 5,134 AT events in 3,503 genes, 2,426 AA events in 2,272 genes, 2,133 AD events in 1,942 genes, 5,134 AT events in 3,503 genes, and 108 ME events in 34 genes (Figure 2A). Accordingly, one gene could express 6 splicing patterns, and ES was the most prevalent one. Finally, 3,993 OS‐SEs from 2,937 genes were figured out and depicted in the UpSet plot (Figure 2B). And ES was also the most common pattern. The volcano plot revealed that most ASEs had a connection with overall survival in MESO (Figure 3A). The top 20 OS‐ASEs in each pattern were shown using bubble plots (Figure 3B–H).

**TABLE 1:** Baseline information of 84 patients diagnosed with mesothelioma

Variables	Total Patients (*N* = 84)
Survival Time, days	
Mean SD	640.01+550.17
Median(Range)	564.50(−8–2790)
Survival State	
Alive	17(20.24%)
Dead	67(79.76%)
Gender	
Female	15（17.86%）
Male	69（82.14%）
Distant metastasis	
Yes	25(29.76%)
No	59(70.24%)
Bone metastasis	
Yes	4(4.76%)
No	8,095.24%)
Stage	
Stage I	10(11.90%)
Stage II	16(19.05%)
Stage III	42(50.00%)
Stage IV	16(19.05%)

Abbreviations: ASEs, Alternative splicing events; OS‐SEs, Overall survival associated splicing events; SF, Splicing factors.

### 3.2 Construction of the predict model

**FIGURE 4:**
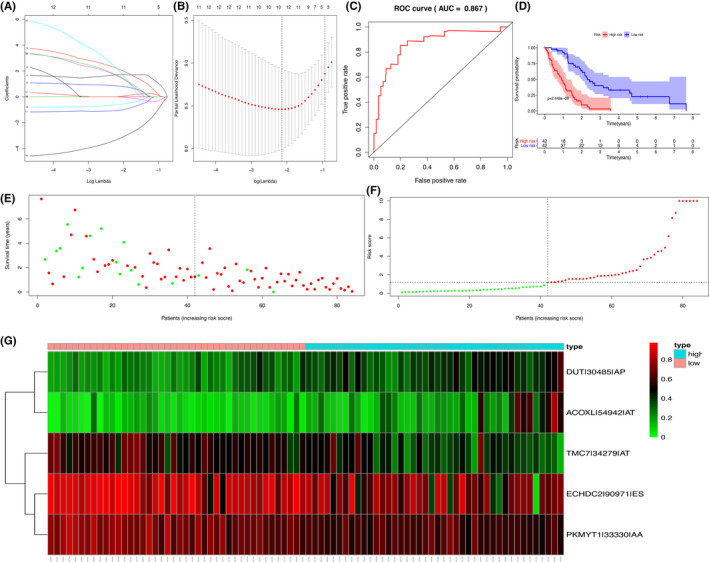
Construction and evaluation of the prognostic model. (A) (B) The lasso regression for screening OS‐SEs; (C) The ROC curve to assess the reliability of the prognostic model (AUC: 0.867) (D) K‐M survival analysis demonstrated that risk score of the model can predict the prognosis patients with MESO; (E) The scatter plot shows clinical status using green and red dots describing survival and death; (F) The risk score of each patient in our study; (G) The expression level of 5 OS‐SEs screened by Lasso regression

In order to restrain features of the prognostic model, we Implemented the Lasso regression method. DUT‐30485‐AP, ACoxL‐54942‐AT, ECHDC2‐90971‐ES, C16orf13‐32921‐ES, TMC7‐34279‐AT, MOK‐29361‐AP, RPL13‐392312‐AD, and PKMYT1‐33330‐AA were finally integrated into the multivariate Cox regression analysis (Figure 4A, B). A decent reliability was shown by ROC curve (AUC: 0.867) (Figure 4C) and Kaplan‐Meier curve also demonstrated a good effectiveness of this prognostic model (*p* < 0.001) (Figure 4D). Scatterplots indicated that patients with higher risk scores had a higher mortality compared to low‐risk patients. That also confirmed the good reliability of the prognostic model (Figure 4E, F). The heatmap visualized the expression level of selected ASEs, in which TMC7‐34279‐AT, ECHDC2‐90971‐ES and PKMYT1‐33330‐AA were high expressed in patients with higher risk scores, while DUT‐30485‐AP, ACoxL‐54942‐AT were low expressed (Figure 4G).

### 3.3 Independent prognostic analysis

**FIGURE 5:**
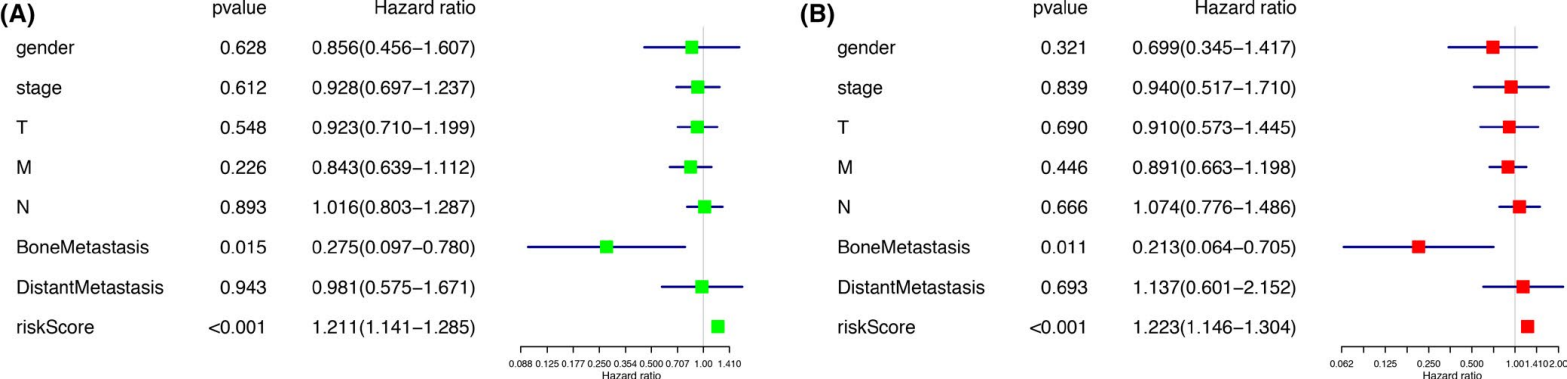
Univariate and multivariate Cox regression analysis for evaluating the independent prognostic value of the risk score: (A) Univariate and (B) multivariate Cox regression analysis verified the risk score to be independent prognostic factor

We performed univariate and multivariate Cox regression analysis, to figure out if the risk score was a predictive factor independently. The independence of the risk score was demonstrated by results of both univariate (HR =1.211, *p* < 0.001, 95%CI (1.141–1.285)) and multivariate (HR = 1.223, *p* < 0.001, 95%CI (1.146–1.304)) Cox regression analysis (Figure 5A, B).

### 3.4 The potential regulatory network of SFs and OS‐SEs, and their bone metastasis correlation

**FIGURE 6:**
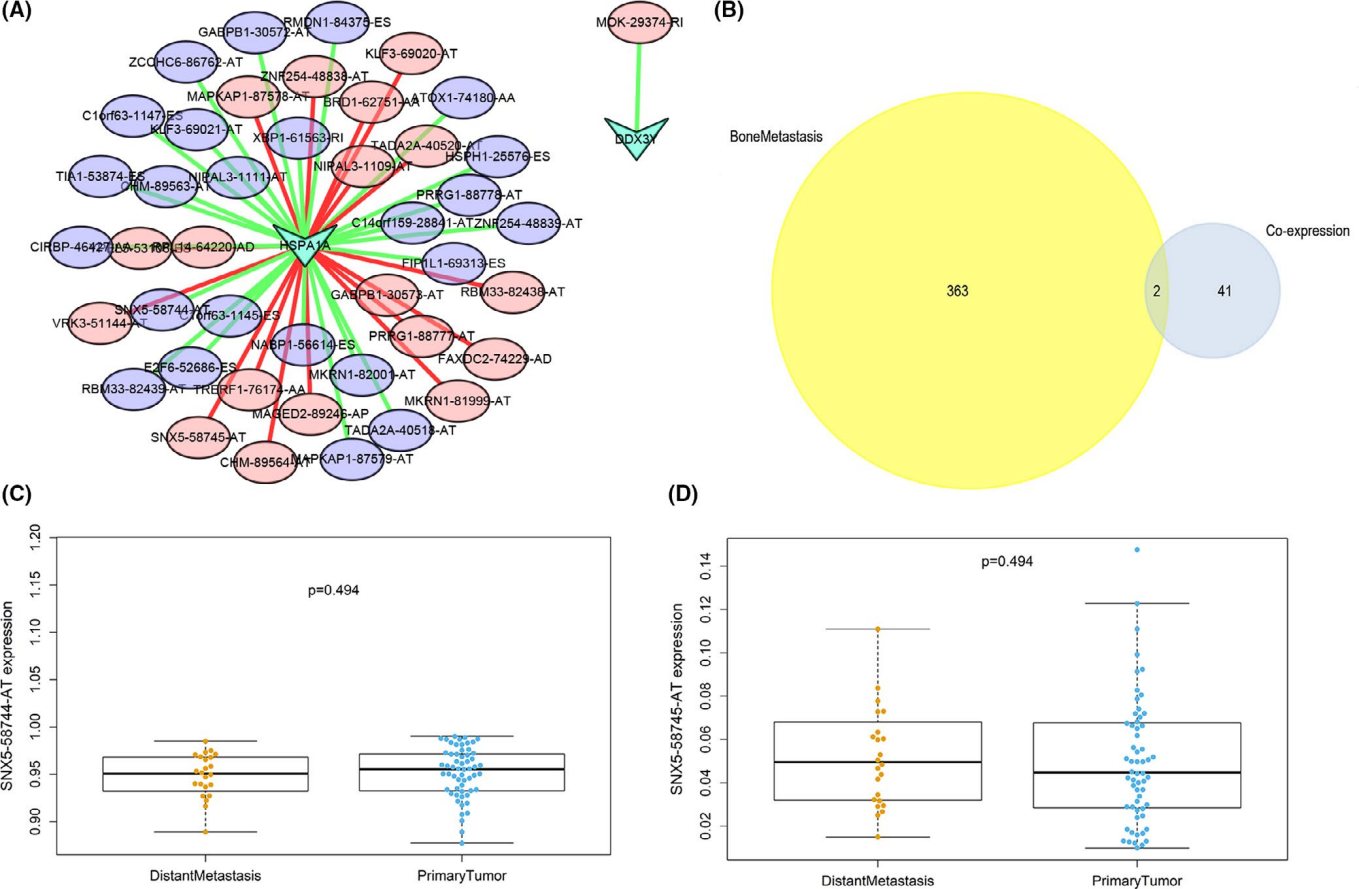
(A) The regulation network of OS‐SEs and SFs; (B) Venn plot OS‐SEs related to bone metastasis; (C‐D) SNX5‐58744‐AT and SNX5‐58745‐AT were correlated with distant metastasis in MESO

According to results of Pearson correlation analysis, splicing factor HSPA1A had a connection with 18 adverse OS‐SEs (red ellipses) positively (red lines) and 24 favorable OS‐SEs (purple ellipses) negatively (green lines). DDX3Y was correlated with 1 adverse OS‐SE (red ellipse) negatively (green line) (Figure 6A). Among these OS‐SEs, two (SNX5‐58744‐AT and SNX5‐58745‐AT) were significantly associated with bone metastasis and were shown in the Venn plot (Figure 6B).

### 3.5 Functional enrichment analysis of OS‐SEs

**FIGURE 7:**
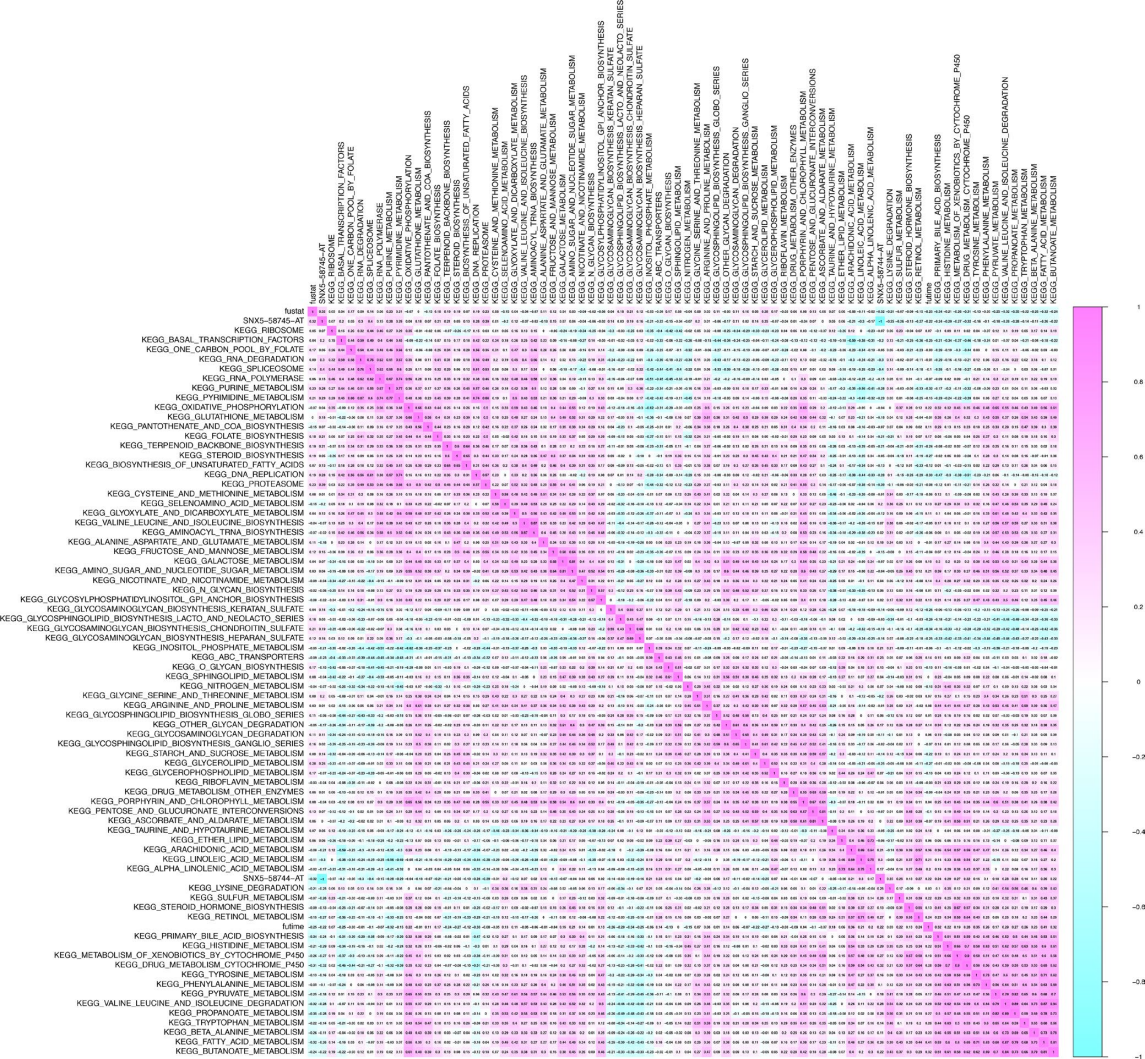
Co‐expression heatmap between OS‐SEs (SNX5‐58744‐AT, SNX5‐58745‐AT) and prognosis‐associated signaling pathways

In total, 185 OS‐related KEGG pathways were figured out using GSVA and the univariate Cox regression analysis. Pearson correlation analysis was conducted to illustrate their co‐expression patterns with SNX5‐58744‐AT and SNX5‐58745‐AT. Co‐expression heatmap showed that the top 3 related pathways were “Spliceosome” (R = 0.400, *p* < 0.001), “Proteasome” (R = 0.390, *p* < 0.001) and “Drug metabolism cytochrome P450” (R = 0.320, *p* < 0.001) (Figure 7).

### 3.6 External validation

**FIGURE 8:**
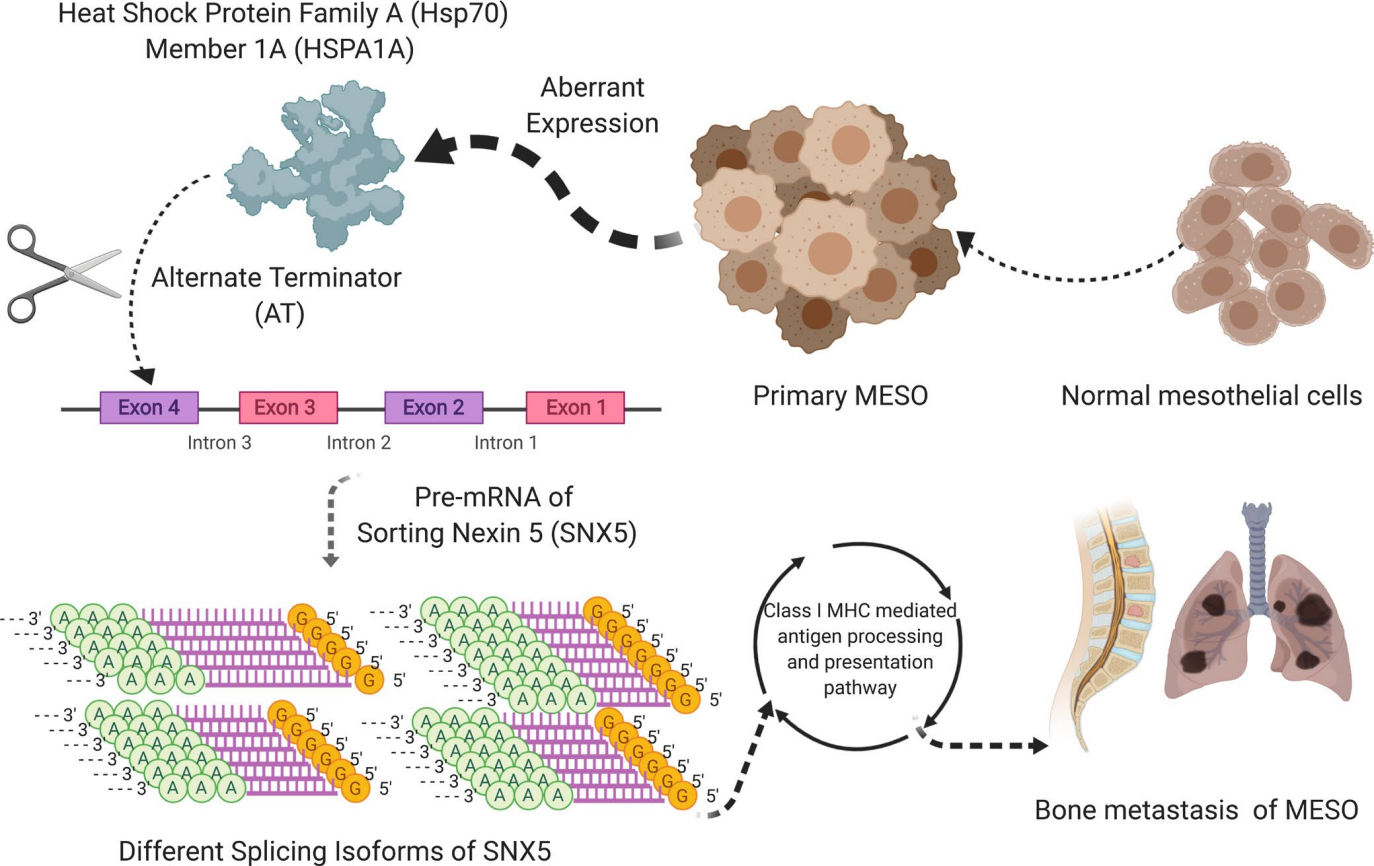
Association of SNX5 with OS and metastasis: SNX5 was significantly associated with OS according to data from GEPIA (A), LinkedOmics (B) and UALCAN (C) datasets; SNX5 was significantly correlated with metastasis in LinkedOmics (D)

The top 3 relative pathways of SNX5‐58744‐AT, SNX5‐58745‐AT were input to Genecard database. Matched pathway name of “Spliceosome” and “Proteasome” in Genecard were “mRNA splicing‐major pathway” and “Class I MHC mediated antigen processing and presentation” while “Drug metabolism cytochrome P450” didn't have other names. Top 5 key genes of each pathway were validated in external databases. Eventually, 9 genes were identified associated with survival and metastasis (Table 2). And results of SNX5 were particularly collected in Figure 8. The overlapped significant genes in all the external databases were CDC20 and SF3B4. In the UALCAN database, SNX5, HSPA1A, BLMH, CCNF, CDC20, SF3B4, CYP3A4 and UGT1A9 were related with OS (Figure S1, B–I) and tumor stage (Figure S1, J–R). And CDC27 was associated with tumor stage only (Figure S1, O). In the LinkedOmics database, all these 9 key genes: SNX5, HSPA1A, BLMH, CCNF, CDC20, CDC27, SF3B4, CYP3A4 and UGT1A9 were associated with OS (Figure S2, A–I), metastasis (Figure S2, J–R) and tumor stage (Figure S2, S–A). Results of GEPIA revealed SNX5, BLMH, CCNF, CDC20, CDC27, SF3B4, and CYP3A4 were associated with OS significantly (Figure S3, A–G). In addition, expressions of CDC27, BLMH, CCNF, CDC20 and SF3B4 were significantly associated with SNX5 (Figure S3, H–M). In the PROGgeneV2, HSPA1A, CDC20, CDC27 and SF3B4 were identified as OS‐associated genes (Figure S4, C–E). According to the analysis of GEO data, BLMH, CCNF, CDC20, CDC27, DDX3Y, SF3B4 and SNX5 were differently expressed between MESO and normal tissue, no matter the specimen type (Figure S5, A–C). Corresponding heatmaps further showed the results in individuals (Figure S5, D–F). To further increase the credibility of the article, we also discussed how to experimentally validate the proposal that HSPA1A could regulate SNX5‐58744‐AT and SNX5‐58745‐AT through “Class I MHC mediated antigen processing and presentation” pathway in the future work (Text S1).

**TABLE 2:** Summary of multidimensional external validation results using multiple databases

	SNX5		HSPA1A		BLMH		CCNF		CDC20		CDC27		SF3B4		UGT1A9		CYP3A4		Results
	N	T	N	T	N	T	N	T	N	T	N	T	N	T	N	T	N	T	
UALCAN	NA	－	NA	NA	NA	↓	NA	NA	NA	↓	NA	↓	NA	↑	NA	NA	NA	NA	SF3B4 high‐expressed while BLMH, CDC20, CDC27 low‐expressed in MESO (Figure S1).
LinkedOmics	↓	↑	↓	↑	↑	↓	↓	↑	↓	－	↑	↓	↑	↓	↑	↓	↓	↑	CDC20 was low expressed in normal tissue; SNX5, HSPA1A, CCNF and CYP3A4 were low expressed in normal tissue and high expressed in MESO; BLMH, CDC27, SF3B4 and UGT1A9 were high expressed in normal tissue and low expressed in MESO (Figure S2).
GEPIA	NA	↑	NA	↑	NA	－	NA	↓	NA	－	NA	↓	NA	↑	NA	ND	NA	ND	SNX5, HSPA1A, SF3B4 were high expressed in MESO while CCNF, CDC27 were low expressed in MESO (Figure S3).
PROGgeneV2	NA	－	NA	－	NA	－	NA	NA	NA	↑	NA	↑	NA	↑	NA	NA	NA	NA	CDC20, CDC27 and SF3B4 were highly expressed in MESO (Figure S4).
CCLE	NA	↑	NA	↑	NA	↑	NA	－	NA	↑	NA	↑	NA	↑	NA	－	NA	↓	SNX5, HSPA1A, BLMH, CDC20, CDC27 and SF3B4 were expressed highly in MESO; CYP3A4 was expressed lowly in MESO (Figure S4).
cbioportal	NA	－	NA	－	NA	－	NA	－	NA	↑	NA	↑	NA	－	NA	－	NA	－	CDC20 and CDC27 were expressed highly in MESO (Figure S4).

Note

“N” was defined as normal; “T” was defined as Tumor; “↑” was defined as a significantly high‐expressed gene; “↓” was defined as a significantly low‐expressed gene; “NA” was defined as “Not available”; “ND” was defined as “Not detached”; “‐” was defined as a gene with no significant difference in expression.

Abbreviations: CCLE, Cancer Cell Line Encyclopedia; GEPIA, Gene Expression Profiling Interactive Analysis; MESO, mesothelioma.

## 4 DISCUSSION

Mesothelioma is an infrequent tumor with poor prognosis. The median overall survival of untreated patients is only 6 to 9 months. Different multimodality treatment approaches, combining surgery, immunotherapy and chemotherapy radiation therapy (RT) are investigated nowadays.^32^, ^33^ However, high frequency of drug resistance immensely impedes the survival improvement of mesothelioma patients.^34^ ASEs were reported to have a significant impact on various hallmarks of cancers, including tumorigenesis, metastasis and drug resistance.^35^ Previous study has identified altered pathways and expression profiles between mesothelioma and other cancers,^36^ however, it hasn't regarded ASEs as biomarkers for MESO and thus the prognostic value of AESs in MESO needs further validation. In this study, we proposed that the splicing events SNX5‐58744‐AT and SNX5‐58745‐AT, regulated by splicing factors HSPA1A and DDX3Y, have an impact on tumorigenesis and bone metastasis through “Class I MHC mediated antigen processing and presentation” and were associated with prognosis of patients simultaneously.

Exons can be spliced constitutively or alternatively, and it is alternative splicing that endow cells to produce different protein isoforms to accomplish multiple biological processes.^37^ To maintain normal physiological function, exons of pre‐mRNAs must be precisely identified and ligated, in a process catalyzed by the spliceosome through two sequential transesterification reactions.^38^, ^39^ Failure in recognition of exon‐intron boundaries or in removal of introns may results in aberrant gene expression, some of which are correlated with diseases.

The spliceosome is a unique protein‐directed metalloribozyme that dynamically assembled from five small nuclear ribonucleoproteins (snRNPs) (U1, U2, U4, U5 and U6) or four snRNPs (U11, U12, U4atac and U6atac), and U5 snRNP is shared by both spliceosomes.^39^, ^40^, ^41^ Generally, spliceosomal assembly initiates when the splice sites (ss) are recognized by the U1 snRNP and the U2 snRNP auxiliary factor (U2AF), forming an E complex. Then the U2 snRNP will be recruited to the branch‐point (BP) to form an A complex, in an ATP‐dependent manner. U4/U6.U5 will subsequently recruited, resulting in the formation of the B complex, which can convert into the catalytically active spliceosomal C complex through a series of structural rearrangements.^42^

The two transesterification steps on pre‐mRNA are catalyzed during the assemble process of spliceosomes. In the first transesterification step, the phosphodiester bonds of the upstream is attacked by the 2’ hydroxyl group of the branch‐point adenosine. This generates a branch lariat and a free 3’ hydroxyl group on the upstream exon. In the second step, the free hydroxyl group attacks the 3’ intron‐exon junction, ligating two exons and releasing a lariat intron.^43^ An array of more than 300 protein factors are involved in the catalytical process, by constituting or regulating the spliceosome.^44^, ^45^ Regulators may function as GTPases, RNA helicases, protein isomerases and so on.^46^ These regulators are all termed as splicing factors (SFs).

Non‐structural proteins such as the serine/arginine‐rich (SR) proteins, are a group of highly conserved regulator and are characterized by a peculiar carboxyterminal domain that highly enriched in Arg/Ser dipeptides (RS domain) and the existence of 1–2 RNA recognition motifs (RRM).^47^, ^48^ Previous studies indicated that the RS domains mainly participate in protein‐protein interactions while the RRMs determine the substrate specificity by sequence‐specific binding mechanism.^49^, ^50^, ^51^ Based on these structures, SR proteins can form a network of protein‐protein interactions by cooperation with U2 auxiliary factor (U2AFs) in the early stage in spliceosomal assembly.^52^ And they also bind to exonic splicing enhancers (ESEs) to promote exon definition by recruiting other splicing machinery or by antagonizing the negative activity of heterogeneous nuclear ribonucleoprotein (hnRNP) recognizing exonic splicing silencers (ESSs).^46^, ^53^ In addition, SR proteins were demonstrated to be dose‐dependent positive regulators of AS through stabilizing the binding of U1 snRNP or activating enhancers.^54^, ^55^, ^56^

While SR proteins generally recognize splicing enhancers, hnRNPs tend to interact with exonic and/or intronic splicing silencers (ESSs and/or ISSs).^57^, ^58^ Previous studies indicated the antagonistic function of hnRNPs to the activity of SR proteins.^53^, ^59^, ^60^ HnRNPs also mediate the removal of large introns through functioning of their cis‐ and trans‐acting binding sites.^61^ U2AF can be recruited to the enhancer and regulates enhancer‐dependent splicing.^51^ When exons are beset in longer introns, interactions across the exon will happen to ensure the binding stability of the U1 snRNPs and U2AF.^62^, ^63^, ^64^ These actions are termed as exon bridging interactions which can only occur to exons between 50 and 500 nt long.^65^, ^66^ Cooperation of these SFs plays vital role in the accomplishment of alternative splicing.

In this study, we extracted splicing factors list from the SpliceAid, a database based on hand curated literature search.^22^, ^67^ In this database, human RBPs were initially extracted from the UniProt database, exhaustive literature research was subsequently conducted and only experimentally assessed RBPs were finally retained.^68^

ASEs play a role in various cancer hallmarks, including tumorigenesis, metastasis, invasion and cancer drug resistance.^69^, ^70^, ^71^ They can be regulated by SFs and impact cancer progression through diverse pathways.^69^, ^72^ For instance, SF epithelial splicing regulatory protein 1 (ESRP1) can regulate expression of CD44 isoforms through AS in MESO and other cancers.^73^, ^74^ To explore the specific regulation mechanism of ASEs and SFs in MESO, we constructed the network between OS‐SEs and SFs. HSPA1A and DDX3Y were significantly associated with 43 OS‐SEs in the network. Among these OS‐SEs, SNX5‐58744‐AT and SNX5‐58745‐AT were associated with bone metastasis significantly.

HSPA1A, as a member of heat shock proteins (HSPs) family, has a dual function in mammals determined by its location. Intracellular HSPs have a cytoprotective/antiapoptotic function and extracellular HSPs have an immunogenic function.^75^ HSPA1A can enhance the cell survival ability, especially in lethal condition, including heat, oxidative and anticancer drugs.^76^, ^77^ Nowadays, mounting evidence regarded HSPA1A as an important biomarker in tumor progression, metastasis and drug resistance of various cancers. Consistently with our results, Nyman et al. demonstrated that depletion of HSPA1A can inhabitant anti‐apoptotic effect and promote oncogenic potential of transcription factor p73 (Tap 73) through regulate different Tap 73 isoforms, which are products of splicing events.^78^ Kasioumi et al. suggested that downregulating HSPA1A promoted the metastasis of cancer. It could, firstly, establish a cancerous environment and subsequently regulate the metastatic process via EMT and migration processes, and eventually triggering anti‐metastatic properties of cancer cells.^79^ Our hypothesis provided a valuable idea of the regulatory mechanism of SFs and ASEs in cancer prognosis and metastasis.

DDX3Y is located within in human Y chromosome and only expressed in pre‐meiotic male germ cells.^80^ It is essential in early stage of germ cell development.^81^ DDX3Y also has an ATP‐dependent physiologically chaperone function in group I intron splicing in the folding of natural RNA substrate.^82^ Knockdown of DDX3Y in neural progenitor cells hindered cell cycle ongoing and promoted apoptosis, subsequently obstructing differentiation.^83^ The quantitative control of DDX3Y protein was exerted by the characteristic DDX3Y transcript variants with long 5’‐untranslated regions (UTRs). Similar 5’‐UTRs exists in oncogene transcripts and other cell cycle related sequence.^84^, ^85^ DDX3Y was indicated to be positively correlated with OS in MESO, as it was negatively associated with the adverse OS‐SE MDK‐29374‐RI.

The parent gene of the identified bone metastasis‐associated OS‐SEs (SNX5‐58744‐AT and SNX5‐58745‐AT) was verified by comprehensive databases. The gene‐sorting nexins (SNX) takes part in protein sorting and membrane trafficking with the ability to bind specific phospholipids and form protein‐protein complexes.^86^, ^87^ SNX5 was demonstrated to have the ability of inhibiting the degeneration of epidermal growth factor receptor (EGFR) in early studies.^88^

SNX5 is an essential factor in EGFR signaling, whose oncogenesis effect has been identified in various cancers.^89^, ^90^, ^91^, ^92^, ^93^ Zhou Q et al. proved that upregulation of SNX5 in hepatocellular carcinoma (HCC) promotes metastasis and poor prognosis via EGFR pathway. And degradation of EGFR was regarded to be a critical role in the process.^94^ Enhanced expression of SNX5 was also exported in thyroid tumor.^95^ Jitsukawa S et al. demonstrated that SNX5 can lessen the tumorigenic signaling in thyroid cancer.^96^ And the knockout of SNX5 gene significantly changed tumor morphology and slowed down tumor growth in nude mice with lung cancer.^97^ Accordance with our results, ASEs in SNX5 was indicated to be the key mechanism of bone metastasis and poor prognosis in MESO.

To further explore the internal mechanism of HSPA1A regulating SNX5‐58744‐AT and SNX5‐58745‐AT, 3 pathways were confirmed according to the co‐expression analysis. And “Class I MHC mediated antigen processing and presentation” have maximum OS‐associated genes. Complexes consisting of antigenic peptides loaded into MHCs are exposed on the surface of antigen‐presenting cells (APCs). These antigens can activate effector T cells, which can exert their cytotoxic activity and eliminate aberrant cells such as tumor cells.^98^ Induction of immune response in late‐stage cancers showed good therapeutic effects.^99^ Cancer cells expressed decreased or deficient MHC class I can escape immunosurveillance and are not powerful enough to activate anti‐tumor immune responses.^98^ In our study, multiple databases showed significant association between the prognosis and key genes in “Class I MHC mediated antigen processing and presentation”. Therefore, we proposed that SNX5‐58744‐AT and SNX5‐58745‐AT, regulated by HSPA1A, play a critical role in MESO prognosis through “Class I MHC mediated antigen processing and presentation”.

To affirm our hypothesis, identify the cell subtype localization of the key genes in the regulatory mechanism.

There are ineluctably several restrictions of this study which should be acknowledged. First, MESO data acquired from public datasets are restricted, which might result in potential error or bias. Secondly, our results were based on bioinformatics and correlation analysis, which only shows the mathematical probability. Molecular biology experiments are needed to confirm the regulatory mechanism. Restricted to the experimental condition, external databases were utilized to primitively verify our hypothesis on different level. And for a mechanism of SF‐mediated AS, the cell subtype localization of key genes should be further identified using immunohistochemical methods. Thirdly, all transcriptome profiling was conducted by the GPL96 or GPL570 platform and all data sets extracted for construction of the prognostic model were from Western countries. Therefore, the conclusion should be used with caution when applied to samples tested using platforms other than GPL96 or GPL570 and patients from Asian countries. Moreover, how these pathways cooperated with each other is still unclear, and future study should focus on this aspect. The positive or negative regulatory relationship among HSPA1A, splicing isoforms of SNX5 (SNX5‐58744‐AT and SNX5‐58745‐AT), Class I MHC‐mediated antigen processing and presentation pathway, and tumorigenesis/bone metastasis of MESO will be validated by biological function assays like gain/loss of function and rescue assays. These direct mechanism assays (e.g. RNA immunoprecipitation sequencing (RIP‐seq)) might offer more rigorous evidence for these potential therapeutic targets and novel prognostic factors in MESO.

## CONFLICT OF INTERESTS

The authors declare that there is no conflict of interests.

## ETHICAL DECLARATIONS

The study was approved by the Ethics Committee of First Affiliated Hospital of Zhengzhou University.

## Supporting information

Fig S1

Fig S2

Fig S3

Fig S4

Fig S5

Text S1

## Data Availability

All datasets used for this study are available from the TCGA‐MESO program and Gene Expression Omnibus database (https://www.ncbi.nlm.nih.gov/geo/) (GSE number: GSE112154, GSE12345 and GSE99070).
