# Supplementary figures and images for: Identification of prognostic and bone metastasis​‐related alternative splicing signatures in mesothelioma
Source: Cancer Med. 2021 May 26;10(13):4478–92. doi: 10.1002/cam4.3977 (PMC8267146; doi:10.1002/cam4.3977)

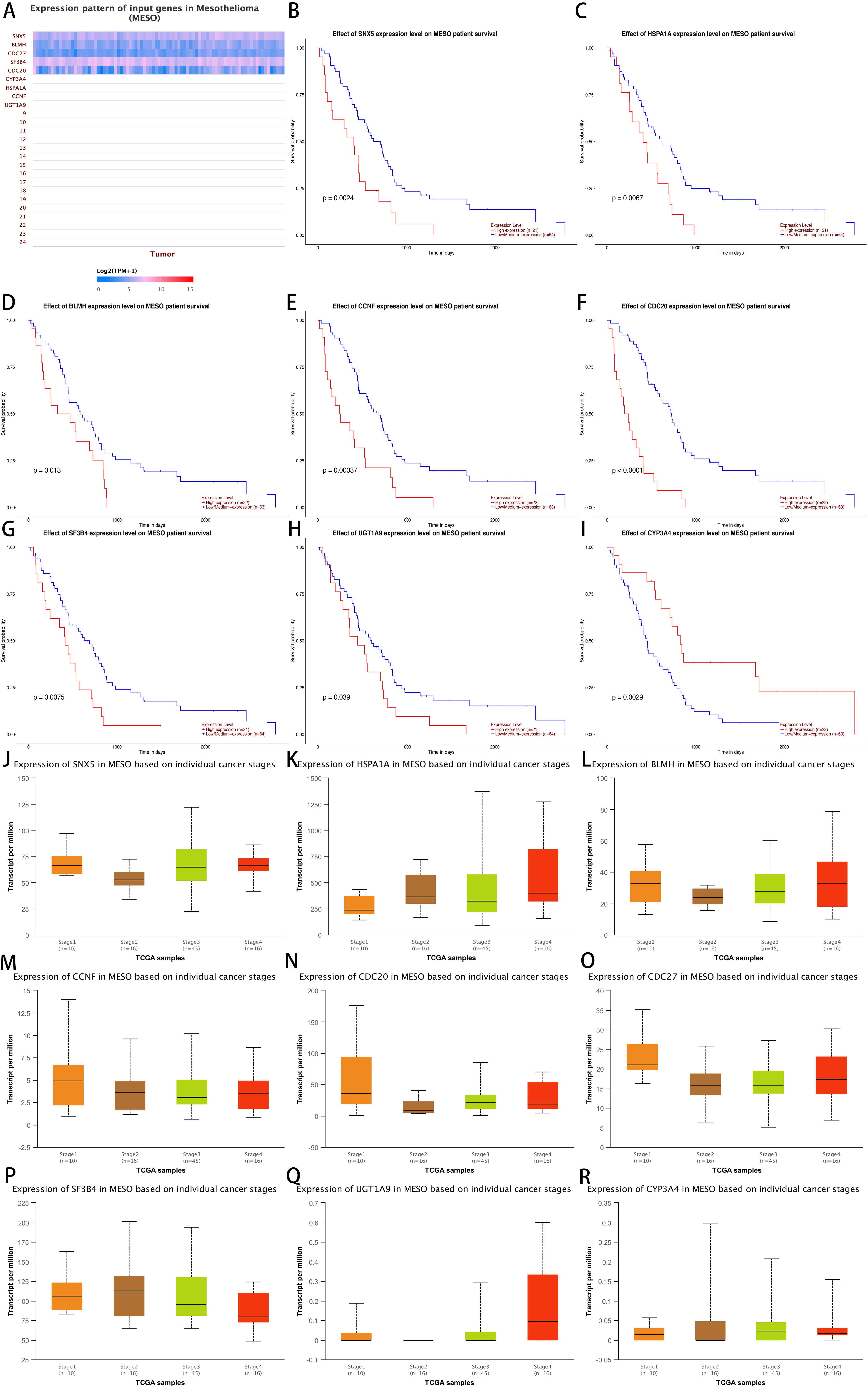

Supplement: Supplementary file 1 — Fig S1 [file CAM4-10-4478-s005.tif]

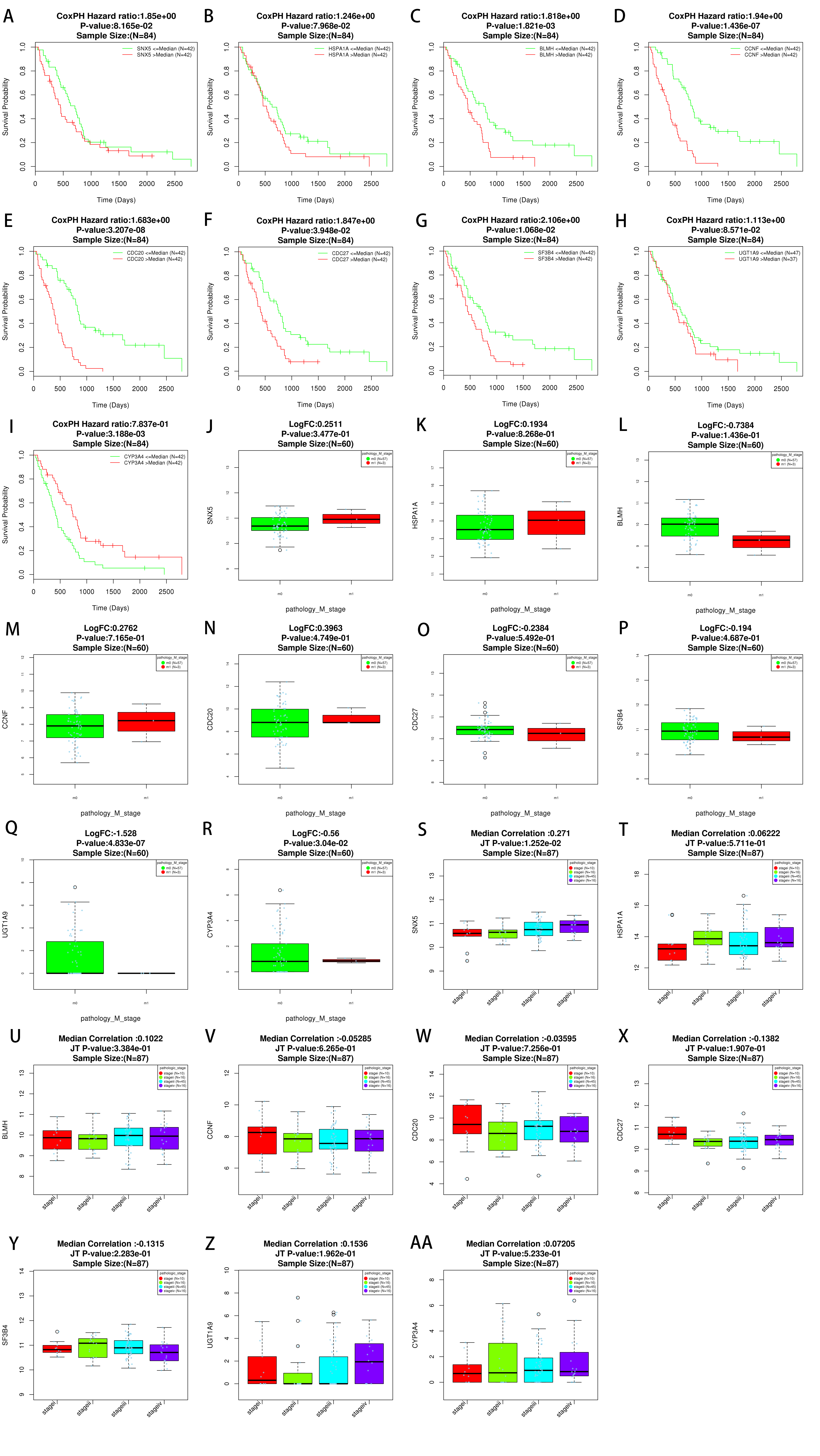

Supplement: Supplementary file 2 — Fig S2 [file CAM4-10-4478-s001.tif]

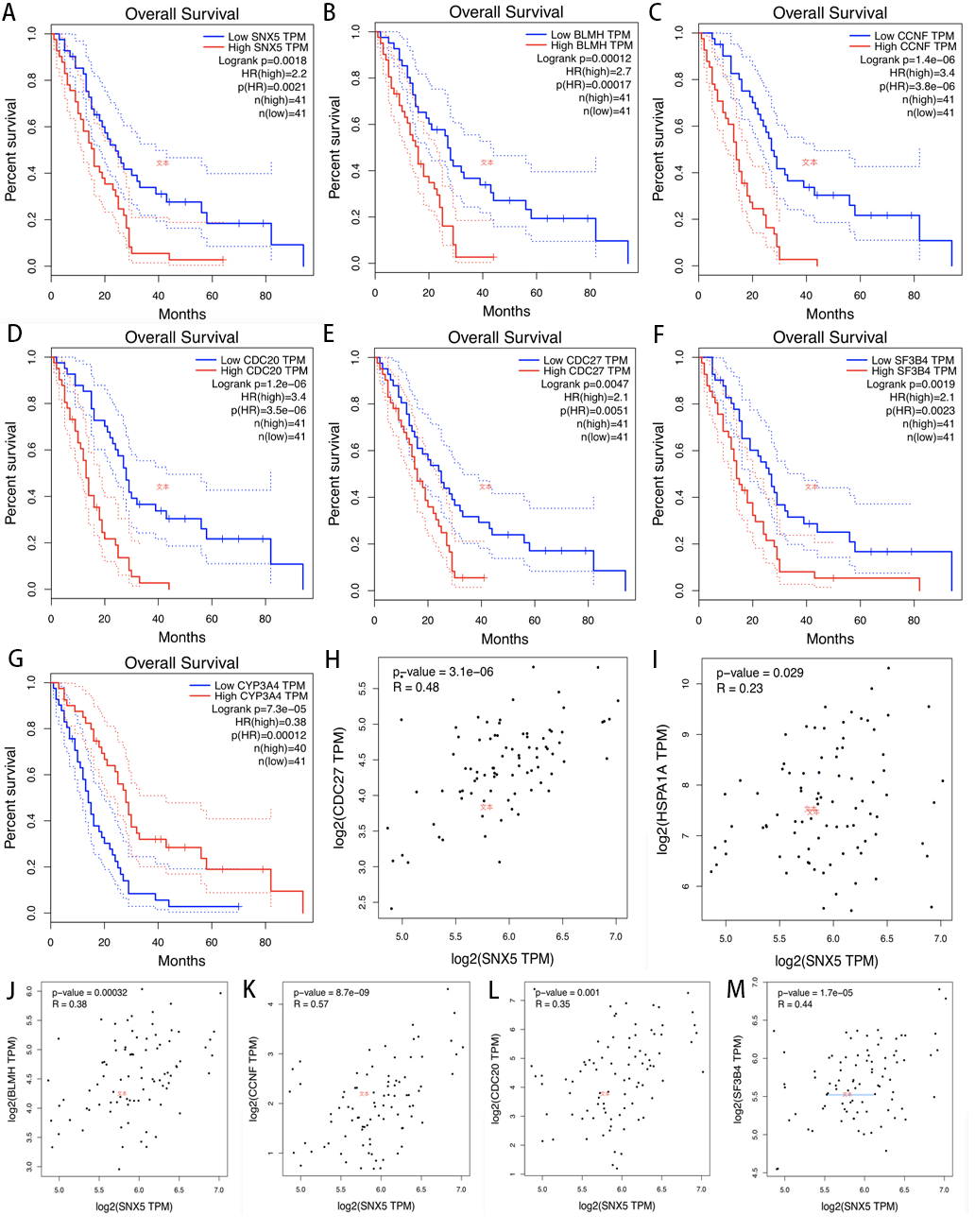

Supplement: Supplementary file 3 — Fig S3 [file CAM4-10-4478-s004.tif]

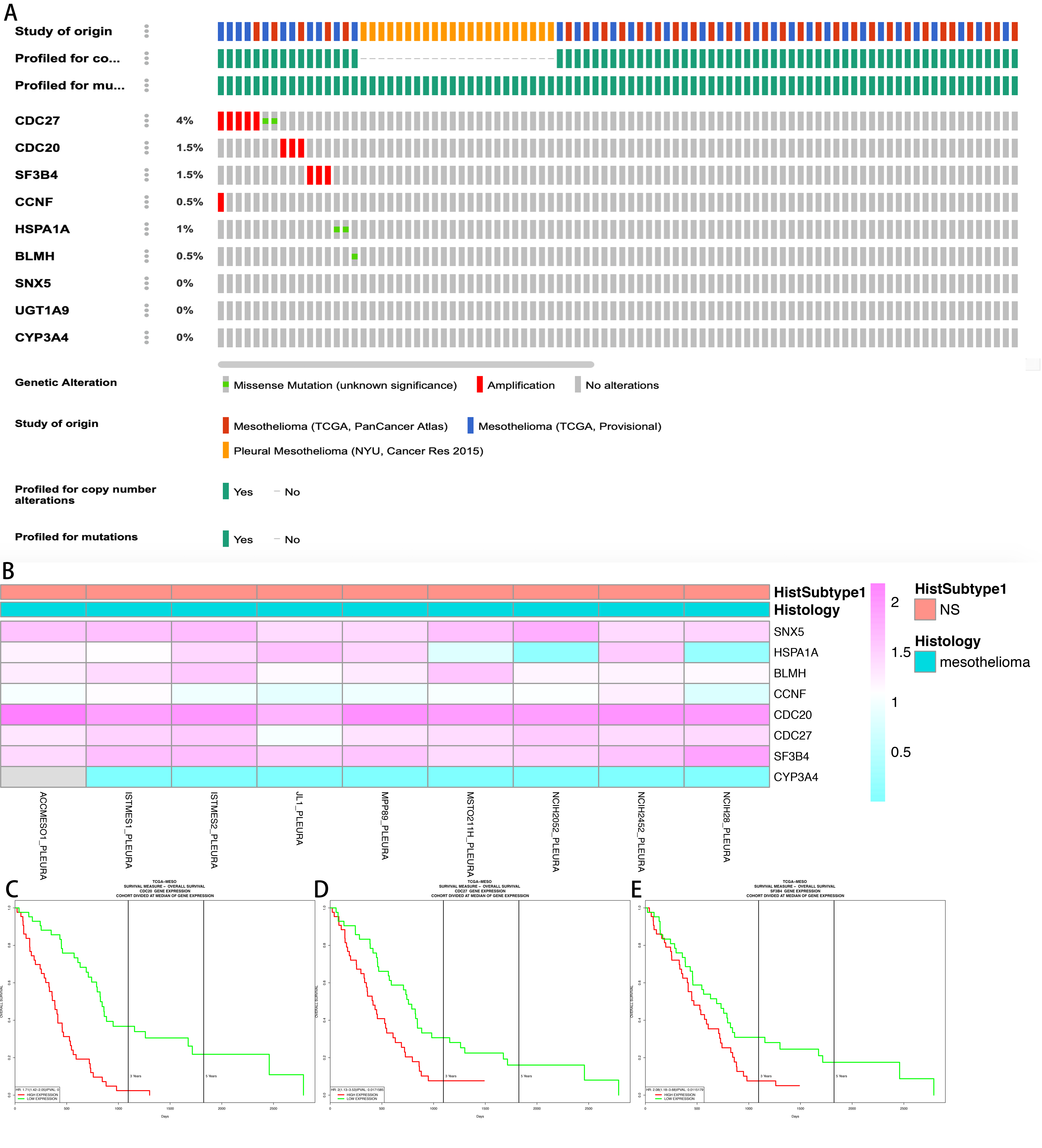

Supplement: Supplementary file 4 — Fig S4 [file CAM4-10-4478-s006.tif]

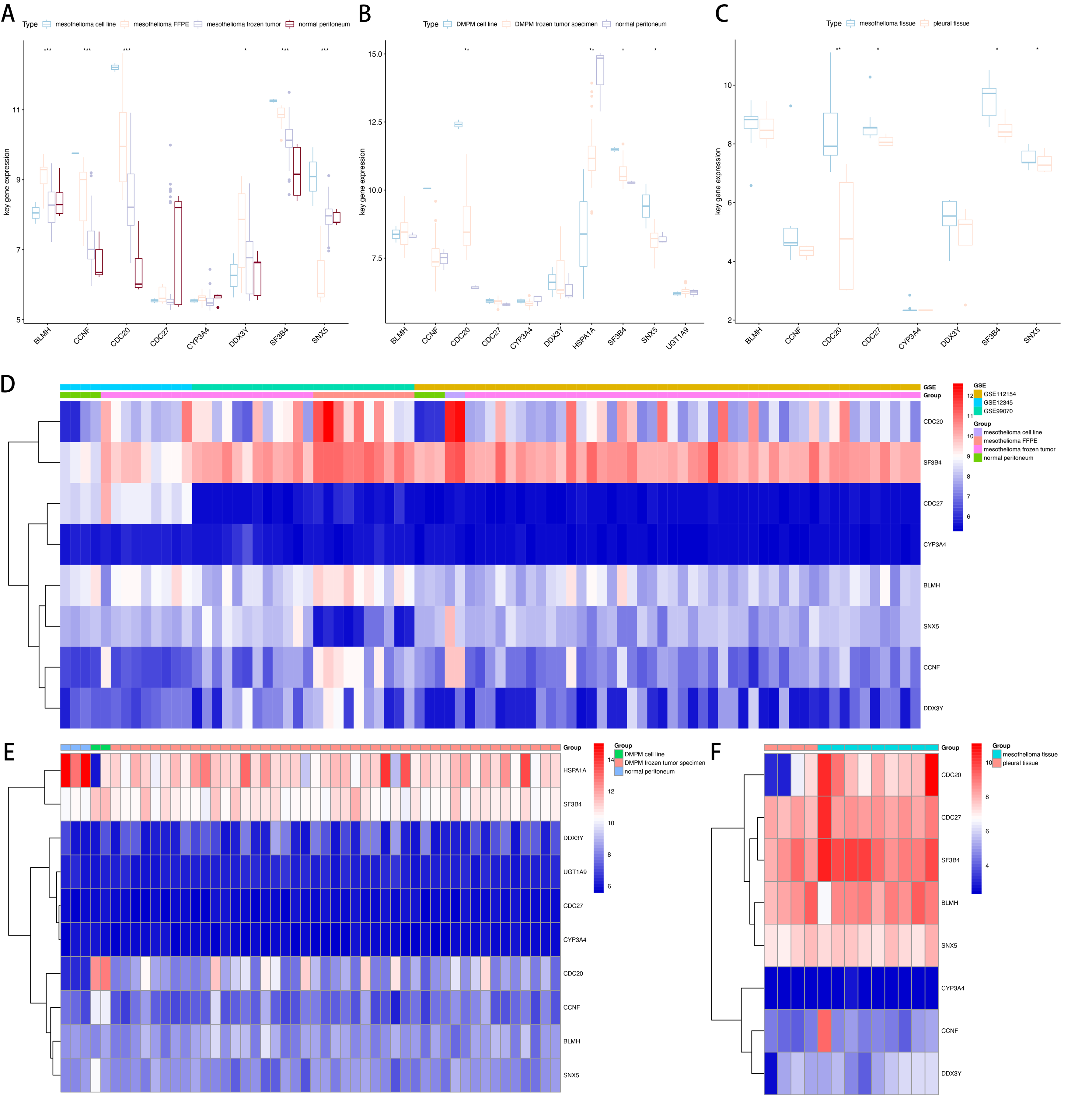

Supplement: Supplementary file 5 — Fig S5 [file CAM4-10-4478-s002.tif]
