# Supplementary material for: Identification of prognostic and bone metastasis​‐related alternative splicing signatures in mesothelioma
Source: Cancer Med. 2021 May 26;10(13):4478–92. doi: 10.1002/cam4.3977 (PMC8267146; doi:10.1002/cam4.3977)
Supplement: Supplementary file 6 — Text S1 [file CAM4-10-4478-s003.docx]

**Supplementary Text 1**

**The research proposal of experimentally validating that HSPA1A could regulate SNX5-58744-AT and SNX5-58745-AT through “Class I MHC mediated antigen processing and presentation” pathway.**

Firstly, we will examine the expression of SNX5 in mesothelial cells and MESO cell lines with different invasive capacity, using quantitative real-time reverse transcription-polymerase chain reaction (qPCR) and Western blot (WB) analysis. Then, lentivirus containing human full-length SNX5, short-hairpin RNA targeting SNX5 or their controls will be constructed and transfected into MESO cell lines. WB will be used to detect the efficiency of the transfection. And expression of cancer cell-derived osteoclast-activating factors IL-6, MCSF, IL-8, RANKL, MMP2, RUNX2 and PTHrP will additionally be measured by qPCR. To detect the mechanism, we will explore the effect of SNX5 in MESO on osteoclastogenesis. Bone marrow-derived macrophages (BMMs) and MCT3T3-E1 will be cultured in a-MEM containing conditioned medium from MESO cells with SNX5 overexpressed, SNX5 knocked or their normal control. Cell viability assay and TRAP staining will be conducted to BMMs. Expression of OPG, RANKL and their ratio OPG/RANKL of MCT3T3-E1 will also be measured. Moreover, to measure the effect of SNX5 in MESO to bone metabolism in vivo, luciferase labeled MESO cells overexpressing SNX5, missing SNX5 or their negative control will be transplanted into nude mice. Bone lesion, tartrate resistant acid phosphatase (TRAP) level and luciferase signals especially in the bone will be detected using micro CT, TRAP staining and luminometer.

SNX5 was demonstrated to be positively associated with HSPA1A in our bioinformatic analysis. To verify this conclusion, RNA immunoprecipitation sequencing (RIP-seq) will be conducted to explore the direct action between HSPA1A and SNX5. HSPA1A expression in MESO cells with overexpressed SNX5, knocked SNX5 or their negative control will also be detected. Furthermore, the HSPA1A inhibitor VER-155008 will be added into the medium of MESO cells overexpressing SNX5, expression of IL-6, MCSF, IL-8, RANKL, MMP2, RUNX2 and PTHrP will be measured by qPCR after incubating for 24 and 48 hours.

To detect the regulation of SNX5 on “class I MHC mediated antigen processing and presentation”, expression of the key factors in the pathway such as CBLB, CCNF, CDC20, HLA-A, HLA-B, HLA-C, LMP7, TAP1 and EZH2 will be detected in MESO cells transfected with SNX5 lentivirus. The silencer of this pathway will also be used to rescue the effect of SNX5 on the expression of cancer cell-derived osteoclast-activating factors.
